# Technical and social issues influencing the adoption of preprints in the life sciences

**DOI:** 10.1371/journal.pgen.1008565

**Published:** 2020-04-20

**Authors:** Naomi C. Penfold, Jessica K. Polka

**Affiliations:** ASAPbio, San Francisco, California, United States of America; La Trobe University, AUSTRALIA

## Abstract

Preprints are gaining visibility in many fields. Thanks to the exponential growth in submissions to bioRxiv, an online server for preprints in biology, versions of manuscripts prior to the completion of journal-organized peer review are poised to become a standard component of the publishing experience in the life sciences. Here, we provide an overview of current challenges facing preprints, both technical and social, and a vision for their future development.

## Unbundling the functions of publication

Science progresses only at the rate at which we can share information with one another. But as any author of a journal article can attest, formal mechanisms of scholarly communication do not always work efficiently and can be subject to biases [1–3]. Peer review takes time: not merely for the reviewer to compile a thorough assessment but also for the editor to find reviewers. In the swiftest case, a manuscript is accepted at the first journal, and the process to eventual publication may take approximately four months [4,5]. However, given that many researchers continue to be evaluated based on the reputation of the journals in which their work is published, authors are incentivised to “aim high” when they select a journal, and it can take several rounds of review (at a single or multiple journals) before the work is approved for publication. It is commonplace for a manuscript to have been submitted to at least two journals on its way to publication, and as a result, the overall peer review process can take years [6].

The sooner a piece of work can be read, evaluated, and built upon, the faster science moves. And by including a greater diversity of thought in the process of science, higher quality final products emerge. Yet, although our system of publication has superficially transitioned from physical print magazines to online websites, the mechanisms and processes of scientific communication are not much faster or more inclusive than they were in the 19th century.

Perhaps the underlying cause for this stasis is the fact that our system of evaluating scientific work—whether for deciding what to read or to whom to award grants and jobs—relies heavily on the reputation of journal titles, and in turn, journal article output is factored into university ranking calculations [7]. Experimenting with new forms of sharing science that are incompatible with publication in traditional venues therefore carries career risks. In addition, many open-science practices (posting lab notebooks, sharing data sets, or conducting replication studies) require significant extra effort for researchers, which is currently not universally prioritised. Therefore, researchers need efficient mechanisms of sharing research that align with current publishing practices while supporting a gradual evolution toward more transparent and efficient communication practices. One small step is to simply share manuscripts publicly at the time they are ready to send to a journal, i.e., by posting a preprint. Preprints are versions of manuscripts made public (often on a preprint server) before the conclusion of a formal (often journal-organized) editorial process. Using preprints to separate in-depth review from the initial act of sharing can increase efficiency while requiring minimal extra work for authors and presenting science in a format that is easily recognized by readers.

Here, we distill what we’ve learned from our work listening to concerns about, and investigating issues surrounding, preprints. We summarize the current state of support for preprinting in the life sciences, discuss extant needs and challenges, and put forth ideas for future developments.

## Why now?

Posting preprints is standard practice in many fields in physics, mathematics, computer science, economics, and other disciplines. Preprints are only now becoming widespread in the life sciences (Table 1), despite a long history of sincere efforts to establish servers in biology by both public and private sectors dating back to the 1960s [6]. Why have they taken off in biology since 2015? We suspect that at least four factors have contributed.

**Table 1:** Developments in preprinting across biomedical and life sciences since May 2018.

May 2018	• Crossref reports that preprints are growing at 10 times the rate of articles [8]
June 2018	• *The Lancet* launches a preprint platform on SSRN [9] • African scientists launch their own preprint repository, AfricArxiv [10]
July 2018	• Europe PMC announces it will now index preprints [11] • PLOS announces they link to the preprint from the published article page [12]
August 2018	• Journal of the American Chemical Society (JACS) permits submission of manuscripts that have been preprinted on arXiv, bioRxiv, and ChemRxiv [13] • ERC indicates 2019 plans to highlight that preprints can be cited in applications [14]
September 2018	• PKP and SciELO announce development of open source Preprint Server system to interoperate with OJS and other SciELO journal systems [15]
November 2018	• Wellcome Trust will require grantees to preprint research where there is a significant public health benefit from January 2020 (now updated from be from January 2021) [16]
December 2018	• ICMJE adds recommendations for medical publishing conduct with respect to preprints [17] • The Israel Science Foundation announces the upcoming launch of ISF Open Research as an open peer review platform for research funded by its programs [18]
January 2019	• EcoEvoRxiv launches as a preprint server for ecology and evolutionary biology [19]
February 2019	• bioRxiv starts rollout of full-text HTML conversion for all preprints [20] • AMRC Open Research officially launches as an open peer review platform for research funded by AMRC member charities [21]
April 2019	• Beilstein Journals host first preprint in their preprint server for organic chemistry and nanotechnology [22]
May 2019	• PLOS has posted 2,500 preprints to bioRxiv through author opt-in upon submission in the first year of the PLOS-bioRxiv preprint-posting partnership [23] • ORCID adds preprint as a “work type” and supports the addition of works using arXiv IDs, enabling authors to document their own preprints in their record [24] • Springer Nature unifies preprint policies on licensing, citation, and media coverage “to encourage preprint sharing” [25]
June 2019	• Research Square’s prepublication platform, In Review, which launched in 2018 with four BMC journals, has expanded and now covers 33 journals and platforms [26] • MedRxiv, a collaboration between CSHL, Yale, and *The BMJ*, launches. The Clinical Science subject category of bioRxiv closes and health-related epidemiology manuscripts are also recommended to be submitted to MedRxiv instead. [27,28]
August 2019	• Open Access India and COS launch IndiaRxiv [29]
September 2019	• PeerJ Preprints stops accepting preprints [30]
October 2019	• CSHL announces Transparent Review in Preprints (TRiP), which will enable journals and peer review platforms to post reviews bioRxiv preprints using Hypothesis [31]
November 2019	• bioRxiv releases information on the server’s practices and usage so far and provides an API [32] • In Review service by ResearchSquare now available for all BMC journals and some SpringerNature titles, with over 9,000 preprints now posted on Research Square [33,34]

Adapted from ASAPbio [35] and additional web search. For developments in May 2018 and earlier, refer to the work by Tennant and colleagues, 2018 [36].

First, in today’s digital world, the idea of composing a manuscript in real-time using collaborative editing tools only to not share it with the community seems increasingly anachronistic.

Second, bioRxiv was positioned effectively within the existing publishing paradigm from the start. Founded by veterans of the publishing industry, John Inglis and Richard Sever, bioRxiv quickly established partnerships with a number of journals. These journals not only agreed to consider manuscripts posted as preprints but also established a direct submission pipeline enabling authors to submit to both with one click. Furthermore, perhaps driven by a competitive publishing environment, editors began to invite submission of manuscripts from preprint servers (discussed below). Preprints now represent an opportunity to publishers, in which previous efforts to share science in this way may have been seen as a commercial threat. Direct submission arrangements and anecdotes about manuscript recruitment offered researchers confidence that posting preprints would not endanger their chances of journal publication. Furthermore, the ownership of bioRxiv by Cold Spring Harbor Laboratory, a credible, nonprofit research institute, likely contributed to its resonance with the community of authors and readers.

Third, many funders have since provided active support and recognition for preprints. Although the NIH has been involved in preprinting through the Information Exchange Groups of the 1960s and Harold Varmus’s 1999 eBioMed proposal [6,37], only recently have many funders voiced support for preprints as a mechanism for applicants and grantees to demonstrate productivity. We discuss these policies in detail below.

Fourth, Twitter created a community that provided visibility to preprints and support to their authors [38]. All of the benefits of preprinting (including discussion, collaboration, visibility, and earlier disclosure) rely on active acknowledgment of preprints by the authors’ community. At the early stages of any movement, adopters will be relatively far and few between, limiting their ability to support one another. Twitter has allowed preprint enthusiasts to connect with one another across institutional boundaries, meaning that even a small number of early adopters can reap the benefits of increased exposure and feedback for their work by sharing preprints with one another.

## Preprints in harmony with journals

In 1966, a cabal of journal editors “outlawed” Information Exchange Groups (the NIH’s photocopy and mail-based preprint exchange platform), fearing that preprints would damage their business model [6]. A representative of the American Association of Immunologists wrote that “Since the preprints are complete publications, there is a real danger that they will reduce the usefulness of existing journals in the field of Immunology and may ultimately supersede them” [39]. Indeed, reports that papers change little between their preprint version to the final published version have caused some to declare that preprints can be the end of the story [40]. Despite the irony that the article reporting this similarity added a section on bioRxiv before its publication in a journal, the more serious issue is that textual analysis may not accurately capture significant changes in meaning. And there is value in evaluation even if the manuscript stays exactly the same: peer review can provide validation as well as improvements.

Perhaps for these reasons, authors continue to use journals even in fields in which preprinting has long been common practice. For example, in physics, 73% of papers on the arXiv can be matched to an article that appears in a journal indexed by Web of Science [41]. Although bioRxiv is younger, the number is similar (67%, [42]), suggesting that neither archive is massively disrupting the journal business.

In fact, preprints are very much complementary to journals, and they offer several tangible benefits for editors and publishers. First, although there is no evidence that the relationship is causal, papers that have been preprinted garner more attention over time [43,44,45]. Preprints allow authors to receive feedback from a broader range of scientists than could be engaged in a typical peer review process. Although a small amount of this feedback appears in the commenting section [46], the majority is communicated elsewhere. For example, a survey of bioRxiv users found that over 40% of authors get feedback via social media, and private feedback from emails and other correspondence with colleagues is nearly as common [32]. In cases in which community feedback on a preprint is incorporated before or during revisions in a journal peer-review process (as we have done for this paper), the version of the paper that is ultimately accepted by the journal will have undergone more scrutiny, likely leading to a higher quality final product. Although the attention each individual paper garners may decline as more and more manuscripts are preprinted (note, for example, that median downloads per bioRxiv preprint in the first month peaked in 2016 [42]), we expect this form of feedback to continue, especially for highly interesting, time-critical, or controversial work that is in the greatest need of additional scrutiny (e.g., see Outbreak Science [47]).

Furthermore, preprints offer an efficient marketplace for papers [48]. Although many editors travel to conferences to invite submission of future manuscripts based on interesting presentations, preprint servers make the manuscripts themselves open to review by anyone in the world. This can enable editors to curate from a wider pool: all preprinted papers, rather than just those actively submitted to their journal. Therefore, it is no surprise that the practice of inviting journal submissions from preprint servers seems to be widespread [49]. *PLOS Genetics* has pioneered the formalization of this process with preprint editors [50] and *Proc B* has adopted the practice as well [51]. Unfortunately, many such invitations may be moot because it is common practice for authors to post the preprint version concurrently with submission to a journal, a process that is facilitated by integrations in both journal and bioRxiv submission systems [27,52]. In order to allow this marketplace of submission invitations to function efficiently, authors can post their preprint a few weeks before journal submission and allow their work to recruit feedback, attention, and editorial invitations. Doing so could help save both authors’ and editors’ time along the way.

Finally, preprints relieve pressure on journals. Authors generally would like their papers to be published as soon as possible, leading some journals to promise shorter peer review turnaround times, perhaps at the expense of allowing reviewers to be as thorough as they would like to be [53]. If authors can instead share a preprint immediately, they are likely to feel more comfortable waiting a bit longer for high-quality, journal-organized peer review.

Journal policies explicitly permitting or even encouraging preprinting have removed much lingering fear of rejection due to prior publication conflicts. Even some long-standing holdouts, notably Cell Press, *JACS*, and the American Association for Cancer Research [54] have updated their policies to be friendlier to preprints. A full list of basic journal policies on preprint archiving can be found at SHERPA/RoMEO [55], more informal lists can be found at Wikipedia [56], and detailed policies on preprint version, licensing, and media coverage policies can be found in Transpose [57].

## Institutional and funder support

Preprints allow researchers to demonstrate their most recent work to prospective and current funders. It is becoming less acceptable to cite work that is “in submission” or “under review” in grant applications: when a manuscript is prepared, reviewers wish to see it and may request the applicant cites a preprinted version [58]. Practically, preprints allow reviewers to judge applicants for funding or promotion by the rigor of their latest science.

In comparison to journals, university policies for the assessment of applications for hiring, promotion, and tenure seem slower to change [59], but there have been bright spots for preprints. For example, in late 2016, NYU Langone Medical Center added language to their promotion and tenure guides to include preprints as a potential research output, and in early 2018, UC Davis added a “preprints” category to the their online faculty evaluation database [60]. UT Austin, The Rockefeller University, and UC Santa Cruz have all added language inviting job applicants for faculty positions to submit preprints as well [60]. Furthermore, preprints may hold value beyond what is codified in formal policies. A survey of hiring committee members conducted by “Future PI Slack” suggests that 10/15 of those surveyed find preprints useful in evaluating faculty candidates [61].

Perhaps the most proactive support for preprints has come from funders, who seemed poised to actively encourage the use of preprints in the life sciences. In May of 2016, the Simons Foundation Autism Research Initiative (SFARI) announced it would change its grant award letter to “strongly encourage” investigators to post preprints and that such papers would be taken into consideration in funding decisions [62]. On September 1 of the same year, these concepts became integrated into the overall Simons Foundation policy, and other funders followed suit, including The Leona M. and Harry B. Helmsley Charitable Trust, EMBO Long-Term fellowships and Young Investigator program, Human Frontiers Science Program, MRC, Wellcome Trust, HHMI, Cancer Research UK, BBSRC, UKRI Future Leaders Fellowship program, CNRS, and the European Research Council [63].

One influential funder policy has been NIH’s guide notice NOT-OD-17-050, which clarifies the NIH’s position on preprints and other interim research products: “The NIH encourages investigators to use interim research products, such as preprints, to speed the dissemination and enhance the rigor of their work … Interim research products can be cited anywhere other research products are cited” [36]. A notable exception, however, is in the use of preprints in post-submission materials [64], which are intended to accommodate events outside the control of the investigators.

Some private funders have gone beyond encouraging preprints to requiring them. Barring privacy concerns, the Chan Zuckerberg Initiative states a commitment to posting preprints prior to peer review [65]. As part of Wellcome’s updated open access policy, researchers working on fields of public health relevance will be required to preprint at the time of journal submission from 2020 [16].

As with all policies, their existence does not ensure they will be enacted. Funders also must develop mechanisms to monitor grantee reaction and compliance. The emergence of technological infrastructure (e.g., links between preprints and published papers, metadata about funding sources, and submission and posting dates), as well as continued dialogue between researchers and funders, is key to enabling these policies.

Some have argued that preprints should be used by funders to achieve open access to the literature in lieu of mandates for open access journal versions [66], but we believe this suggestion is not viable. Because peer review changes papers (often for the better) such a plan would create a two-tier system with some readers having privileged, paywalled access to the more definitive journal versions of papers, and the remainder left with access to other versions that might be outdated. Plan U could become an acceptable substitute for universal open access only if all journals and preprint servers permitted archiving postprints (i.e., versions of manuscripts accepted for journal publication) and/or all submitted version of manuscripts. Currently, bioRxiv does not allow preprint publication after acceptance, and many journals prohibit the publication of preprints that incorporate comments from the peer review process [67].

## Technical issues

At present, preprint servers lack the technological infrastructure that could help them to realize their full potential. Addressing such challenges could make a large impact on how preprints are used and discovered.

For example, authors who have previously read a preprint often wish to quickly find out how it has changed upon the posting or publication of a subsequent version. Currently, neither preprint servers nor journals systematically present a summary of the changes made. Some users already make version notes when posting a revised manuscript to bioRxiv; making this more standard practice might involve enabling authors to submit a short piece of text to journals as well, similar to a conflict of interest disclosure or author CRediT declaration. Once this is complete, it would be natural for journals to provide a link back to the preprint version, which would present a more complete picture of how a manuscript evolved over time. Some journals already provide this backwards link—including *Biophysical Journal*, *Plant Direct*, and *PLOS* titles [68–70]. Formal inclusion of preprint information in the XML representing the manuscript’s version history would help: this recommendation for the JATS schema is currently pending decision [71]. Alternatively, researchers may wish to include a link to their own preprint in the final version (as we do here), where the journal policy permits this. Preprints could also be better supported by reference managers with features that would allow users to link preprints to later versions (whether revised preprints or a final journal version) and receive updates when subsequent versions are available online.

Change is needed in search tools, too. For example, preprints could be linked from PubMed and PubMed Central. (Note that this is effectively being done for papers in *F1000 Research* and associated platforms such as *Wellcome Open Research*. Once these papers pass peer review, they appear on PubMed Central along with their date-stamped first version.) This helps to establish a record of what work was done when, irrespective of delays imposed by the peer review process, which is key to determining priority of discovery. Europe PMC already indexes preprints and has implemented links between the preprint and published version of the same piece of work, though improved metadata could facilitate further search and tool development [11,72].

Beyond the basic metadata about a preprint, open access to the data detailing interactions with each preprint would enable innovation around how the latest science is discussed. For a recent effort to understand Twitter interactions with and downloads of preprints posted on bioRxiv, content metadata was derived by scraping the bioRxiv website [42]. In the absence of an official bioRxiv application programming interface (API), these authors and others had developed their own tools (including an API, command line tool and Python wrapper) to source and interact with bioRxiv content data. Since the publication of the analysis, bioRxiv released its own API [73].

Addressing the technical issues detailed above may help more people find and interact with preprints. As we will discuss in the next section, the low discoverability and perceived legitimacy of preprints is at the root of several more complex social problems.

### Social issues

Today, preprinting is treated as standard practice—or at least supported to a considerable degree—in some life science communities, such as neuroscience, bioinformatics, evolutionary biology, and ecology ([42]; see also subject-specific initiatives like “Peer Community in” [74] and servers hosted at OSF Preprints [75]). Other subject areas have less experience and thus may have lower awareness of the actual benefits and issues. In addition to new servers [28,36,76], several new research categories have been added to bioRxiv in recent years—clinical trials, epidemiology, paleontology, pathology, and pharmacology and toxicology (note their absence in older literature [42,46] and that both clinical trials and epidemiology are now served by medRxiv). This freshness demands and enables considered discussion of important issues so that the most beneficial practices surrounding preprinting can be cemented as cultural norms. A recent consultation highlighted that researchers were often unable to cite case studies of the benefits of preprints [38], and so continued productive adoption may require increasing the number and visibility of shared real-life experiences with preprints, such as those collected by We Support Preprints [77].

### Licensing

Although open access to scholarly literature has been discussed for decades, its original meaning has been diluted. The Budapest Open Access Initiative defines it as “free availability on the public internet, permitting any users to read, download, copy, distribute, print, search, or link to the full texts of these articles, crawl them for indexing, pass them as data to software, or use them for any other lawful purpose, without financial, legal, or technical barriers other than those inseparable from gaining access to the internet itself” [78]. Today, the majority of articles on PubMed Central, although “free” to read, are not actually open for reuse. Articles not in the OA subset cannot be downloaded in bulk, restricting access to text and data mining [79]. Even if that bulk download were available, their licenses do not permit reuse.

Because authors are directly in control of licenses on preprints, they have an opportunity to create a more open corpus of literature. However, most authors on bioRxiv are choosing restrictive licenses [80] amid widespread confusion about what they mean and a misconception that journals prohibit the use of certain licenses for preprints [81]. In reality, we are aware of no publishers that currently enforce this policy. In contrast, an influential funder, the NIH, has recommended the use of CC BY [82]. More education and guidance for authors is needed, e.g., within the preprint submission process itself. Ideally, however, co-authors would have an informed discussion about the license to choose for their preprint before submission.

### Permitted versions

The term “preprint” can describe many different versions of a manuscript, ranging from drafts shared for feedback well before journal submission to manuscripts ready to be accepted by a journal. However, journals differ in their policies regarding which versions of manuscripts under consideration may be posted, with some of them prohibiting the posting of preprints after initial submission. These policies may be rationalized by a sense of journal ownership of the peer review process, but, in fact, they prevent scientists from sharing improvements drawn from diverse sources—their own additional experiments and analysis, feedback from colleagues with whom the manuscript was privately shared, comments on the preprint server itself, and input from social media and preprint-specific feedback platforms (including preLights, PREreview, biOverlay, and Peer Community in). Adding to the confusion, preprint servers differ in their own policies for manuscript deposit; in many disciplines (canonically, arXiv) preprint servers also host postprints. In the life sciences, PubMed Central, complemented by institutional repositories, fulfills this need, and bioRxiv hosts only preprints, not postprints. However, other platforms can host biology postprints, e.g., OSF Preprints.

### Scooping

A common fear cited as a barrier to preprinting is “getting scooped.” Researchers may feel this has happened when a competing research group publishes highly related work without crediting (i.e., fairly citing and discussing) their own preprint. As a consequence, their work receives less attention and recognition, and if the work is still unpublished, this can mean publication in a “lower” journal. Scooping can of course occur in the absence of preprinting when work is discussed at conferences, submitted to journals, or included in funding applications, or simply by coincidence of two groups working in a related area. Fears of scooping may be particularly acute in fields with “a well-defined objective” such as structural biology, although the actual consequences may not be as severe as feared [83]. Preprints do in fact offer some protection against scooping by providing a public timestamp of a claim [84,85]. Nevertheless, the nuances of the timeline of priority claims are likely to be overlooked by casual readers. Approximately 2% of respondents in bioRxiv’s survey report having suffered a loss in ability to claim priority, or to publish in the journal of their choice, as a result of having published a preprint [32].

It stands to reason that scooping fears are most acute when the stakes are high and careers are on the line. However, fears about scooping—and the secrecy that accompanies them—cannot be neatly divided by generations because it’s rare for a group of co-authors to be homogenous in years of experience.

Fear of scooping impacts not only researchers’ willingness to share preprints at all but also whether they are willing to share auxiliary materials that are normally shared as a condition of journal publication. For example, communities have yet to come to consensus on whether authors should be obligated to share reagents or strains after posting a preprint. In a future world where preprinting is universally regarded as a respected disclosure, ethical standards of disclosure should match those associated with journal articles.

### Preprints and the media

One of the major arguments against the use of preprints is concern about exposing the public to unverified information, especially in fields in which public interest is high, such as medicine [86]. For example, one of the Altmetric top 100 papers on 2016 was a bioRxiv preprint linking cell phone radiation to cancer in rats. Some media outlets reported on the paper without mention of its preprint status [87], whereas others provided more measured coverage, including a critique of previous media reports [88].

As preprints rise in popularity, it is important that all readers and especially journalists have access to information about what a preprint server is and the screening checks (or lack thereof) that it employs. Many preprint servers (including bioRxiv, medRxiv, and PeerJ Preprints) display a disclaimer explaining the nature of preprints and the fact that they have not yet been “certified” by peer review.

The reaction of a community via public commenting can also be enormously helpful. Although the majority of feedback on preprints comes via mechanisms that is harder to tie to the preprint itself (or perhaps not publicly visible at all [32]), public commenting sections enable readers to gauge community reactions at a glance. The presence of links to related research documents, such as data and code, are also an important signal of trust for researchers [89]. Although the general public may not recognize these indicators, scientists reading preprints can use them to publicly raise concerns that will benefit all readers.

Moving forward, it would be helpful to provide all readers with information that outlines the level of scrutiny and/or acceptance by experts that an individual research output has received and for journalists and other public commentators to critique accordingly.

### Curation and evaluation

As the production of scientific outputs continues to accelerate, both as a result of a growing number of researchers and their increasing willingness to share, we will need new ways of dealing with information overload. Although a glut of publications may feel like a 21st century problem, thinkers since Seneca have lamented the overabundance of information, and scholars have progressively developed tools to help organize and filter it [90].

Currently, readers report finding preprints by searching for keywords (note that multiple preprint servers are indexed on EuropePMC, OSF Preprints, and Google Scholar). They also report being alerted to interesting work on Twitter. The first strategy is directed by subject area but not interest, and the second by interest but not necessarily subject area. Ultimately, we will need more efficient ways to combine both search criteria in a single stream, in much the way that journal title is presently used (rightly or wrongly) to help parse search results in PubMed. Rxivist is one such tool that marries current interest and subject area [42], and we are collecting more curation projects at reimaginereview.asapbio.org. We believe that this emerging space will become an essential component of the preprint ecosystem.

Curation of interesting or highly respected preprints can also improve their usefulness in evaluating scientists for jobs and grants. Although journal name (and Impact Factor) are flawed proxies for judging the quality of a work [91], they save reviewer time by quickly communicating information about a paper’s selection process. Such proxies are not essential in the late stages of an evaluation process when candidates have been whittled down to a short list and reading their full outputs is a manageable task. However, the process of shortlisting candidates requires more time-efficient indicators of research quality than reading the content itself. Shortly after publication, such indicators may include the level of authors’ transparency and openness, endorsements from peers, and assessments of creativity. In the longer term, established reproducibility or replicability and impact on science or society can also be assessed [92]. Preprints offer the opportunity to evaluate researchers based on their most recent work, but candidates may need to accompany them with indicators that distill community reactions in the short-term, such as downloads, citation counts, constructive preprint comments, and other endorsements. Despite existing limitations, multiple reports suggest preprints are already helping early career researchers to secure their next research position [61,77,93]. Improved practices for filtering, curating, and signaling interest in preprints can further promote this phenomenon.

## The future of preprints

Although the rate of preprints appearing on bioRxiv is increasing exponentially, they still comprise less than 3% of the monthly output of PubMed [94]. Because the majority of the potential growth in the use of preprints lies ahead, research communities must ensure that such use is productive to both science and scientists.

### Who’s at the table?

With reduced gatekeeping mechanisms, preprints could be a mechanism for sharing and consuming the latest science irrespective of social hierarchies. We must ensure that preprint infrastructures and social mechanisms develop with issues of diversity, equity, and decolonialization of scholarship in mind [95,96]. Who can contribute to the preprinted literature? Who benefits from posting a preprint? Who can read, consume, and use information in preprints? As preprinting continues to grow in biology, we must bake these questions into every discussion.

The growing adoption of preprints in biology is being largely driven by researchers in North America and Europe: of the top 100 institutional affiliations ranked by number of preprints posted to bioRxiv until December 2018, only 6 are located outside these regions [42], whereas the 10 institutions affiliated with the most preprints are in the United States and United Kingdom alone [32]. Researchers who feel comfortable posting a preprint are likely to be those who feel less threatened by the “scooping” concerns identified above, which may be affected by perceived and actual competition for recognition, funding, and career positions. On the other hand, preprints may be seen as a cost-effective way to disseminate work particularly in more resource-constrained environments [97]—the advantages and disadvantages of preprints for scientists operating outside the European and US funding context warrant further investigation.

Reflecting on the “scooping” concerns listed above, we should consider how preprints could offer appropriate recognition and support for creators of openly shared work. Indeed, some researchers report only being rewarded with funding and jobs when they are authors of (high-impact) journal articles and not for reuse of their open data sets [98]. Therefore, it can be difficult to argue that the researchers producing the primary data sets should share these openly, let alone rapidly with a preprint. This issue does not relate to the development of new tools and methods—in this case, researchers report valuing the immediate usage, testing, and feedback that preprinting these resources provides.

Once work is shared openly, it is important to address how widely it is seen. Twitter is a major driver of attention on preprints, and social connections between preprint authors and readers raise visibility in the absence of dissemination through journals. Thus the visibility of preprints is strongly influenced by the authors’ existing network “connectedness” and therefore is vulnerable to the same underrepresentation issues we face elsewhere in science. There have been several initiatives to increase the visibility of underrepresented scientists (including VanguardStem and 500 Women Scientists [99,100]); following suit, SBotLite is a new Twitter bot that retweets preprints posted by female first authors in the hope of raising their visibility [101]. Ensuring that the dissemination of preprints does not mimic or perpetuate diversity issues in science, technology, engineering, and mathematics (STEM) requires continued investment in initiatives to counteract and mitigate existing attention biases.

### Beyond the article

Some have expressed concern at the roughly 35% of preprints that do not go on to be published in a journal, believing that these preprints must be of low quality [102]. Alternatively, these outputs could reflect work never destined for a journal that would have otherwise not been shared or work that the authors have chosen not to submit to a journal. Such products include negative results, preliminary findings, methods and protocols, and short reports from projects that could not be completed (e.g., because funds or a training period ran out). All of these products are valuable, and all could be in principle posted on a preprint server. bioRxiv, however, does not allow “theses, dissertations, student projects, recipes and simple protocols,” nor review or policy articles [27]. It does, however, have specialized sections for contradictory and confirmatory work, though they are seldom used. As of the time of writing, the Contradictory and Confirmatory Results sections together make up less than 3% of the articles on bioRxiv [103].

These low usage rates suggest that preprints alone are not likely to be a solution to publication bias; our current incentive system does not sufficiently reward investments of energy spent writing up contradictory or confirmatory findings in the format of a journal article. Some of this effort, e.g., carefully assembling a methods section, is necessary to reproduce the work and must not be compromised. But other tasks, like putting the work in context with an introduction or interpreting the findings in a discussion, is less useful to specialized readers, who are the likely audience for contradictory or confirmatory findings anyway. In fact, those readers do not need the element of a narrative (often constructed post-facto) that ties together figures in a traditional paper. In these cases, a single figure (or even a micropublication, defined for these purposes as a statement with attribution [104]) would suffice.

There is presently an expectation that all products appearing on preprint servers are more or less complete articles. This helps to promote an image of the preprint server as a destination for high-quality work and helps to facilitate some very positive behaviors, such as the soliticiation of submissions by journal editors. However, this norm reinforces a culture in which research is shared relatively late in the process and also feeds some behaviors that are less desirable, such as counting the number of papers on a CV as a measure of productivity without assessing their contents. Although this practice makes little sense, it is a real concern, as evidenced by the fact that the Medical Research Council worded its preprint policy to discourage researchers from “salami slicing” their preprints into many smaller units for the purpose of gaming the system by gaining a higher publication count [105]. It is not useful to science for researchers to split one story into multiple parts purely to game the evaluation system; however, given the deeply complex and technical interdisciplinary work that is now often combined into a single 1,500-word article, there is clear value in ensuring each finding is comprehensively described. If posting single figures or smaller increments of work were to become standard practice, all research results could be communicated faster and with adequate methodological description to ensure reproducibility. Those ultimately destined for a journal could be assembled into an article when the authors felt ready. Another benefit of micropublications is that they enable peer review on a more atomic level. In an environment in which papers result from the collaboration of many different specialized experts, there may be situations in which no two or three reviewers have sufficient expertise to cover every figure panel.

Despite the apparent benefits of micropublications and preprints, both technical and social innovation is required to address open questions. Namely, how can science be shared in varying orders of detail, complexity, and review status over time, from first observation of a result to acceptance of a generalized finding into broader understanding? Which research outputs (data, code, methods) are useful to embed in a narrative article? For which of these outputs is subsequent filtration and curation valuable? Ultimately, where it is most useful to invest resources in coordinated peer review, journal production processes, and dissemination of findings to nonspecialist communities? Regardless of when or how preprints fit into this picture, we should strive to ensure that research integrity is rewarded, discovery is accelerated, and the publication process is more inclusive and equitable.
